# Achilles’ heel: elderly COVID-19 vaccination policy in China

**DOI:** 10.1186/s12961-024-01155-1

**Published:** 2024-08-05

**Authors:** Ziru Deng, Karen A. Grépin

**Affiliations:** School of Public Health, Li Ka Shing Faculty of Medicine, The University of Hong Kong, Patrick Manson Building, 7 Sassoon Road, Pokfulam, Hong Kong

**Keywords:** COVID-19, Elderly vaccination, Policy analysis, Multiple streams framework

## Abstract

**Background:**

Despite high overall COVID-19 vaccine coverage, the continuously low elderly vaccination rate in mainland China remains a dangerous threat as the country shifts away from its zero-Covid policy. This retrospective study uses the Multiple Streams Framework to examine how macro-level factors may explain poor elderly vaccination outcomes.

**Methods:**

We performed a thematic analysis of qualitative data obtained from 95 official press conferences from October 20, 2020, to February 27, 2023, vaccination-related policy documents, and media coverage, using both inductive and deductive coding approaches.

**Results:**

Our findings suggest that in the problem stream, elderly vaccination was not a “focusing event” during the initial vaccine rollout, resulting in delayed outreach to this population. Additionally, ideologically driven complacency and discrepancies in top-down implementation undermined elderly vaccination in the political stream. In the policy stream, precautious and ambiguous statements, inconsistent policy content, radical shifting media messages, and less age-friendly digital technologies also affected elderly vaccination.

**Conclusions:**

The poor convergence of the three streams led the elderly to be the Achilles’ heel of China’s COVID-19 containment strategy. Future studies should focus on priority identification, adoption of enforcement measures, and timely and effective policy dissemination. The empirical lessons from China can inform and optimize elderly vaccination policy design and implementation in the post-pandemic era.

## Background

By the end of 2021, most former “zero-COVID” countries had lifted restrictions and phased out elimination policies due to the widespread availability of vaccines and the high costs of maintaining this approach. However, it was not until January 8, 2023, after issuing new guidelines, that mainland China ended its pursuit of elimination and reopened [1]. Although China had invested enormous resources and systems to facilitate its mass COVID-19 vaccination program since late 2020, and despite the overall high vaccination rates among the general population, the disparity in its elderly vaccination rates was a grave risk during its reopening. This unusual pattern of elderly vaccination also set China apart from much of the rest of the world. As of July 2022, 19 months after the vaccination program had commenced, only 50.7% of the elderly over 80 years old had received the first dose of vaccination [2], while Japan and the United States had already achieved vaccination coverage rates of 92.7% and 91.7%, respectively, among individuals over 65 years old during the same period [3, 4]. The low and sluggish uptake by the elderly, who are at the highest risk for severe illness, hospitalization, and death from COVID-19, posed a significant threat to the resilience and efficacy of China’s healthcare system.

Perplexingly, China exhibited one of the lowest COVID-19 vaccine hesitancy rates worldwide [5]. Surveys conducted prior to the national mass vaccination campaign showed that elderly citizens displayed a willingness rate of 79.1% to accept COVID-19 vaccines [6]. In April 2021, when the vaccination program for the elderly began, over 90.6% of Chinese adults expressed a strong willingness to be vaccinated, surpassing rates in many other countries [7]. However, the willingness rate among the elderly to accept COVID-19 vaccines was approximately 5% lower than that of individuals aged 18–59, which contrasts with trends observed in the US, France, and other countries [6, 8]. Moreover, trust in government and political attitudes have been found to affect vaccination uptake. Studies in the US and France found that attitudes toward COVID-19 vaccines were significantly correlated with political partisanship and engagement with the political system [8, 9]. Similarly, distrust in government decisions and actions related to COVID-19 containment has influenced public opinion and the intention to receive vaccines in South Korea [10]. Nevertheless, despite this common trend, China’s reputation for strong executive power and a compliant population makes the comparatively low and persistent lag in the elderly COVID-19 vaccination within the country a distinctive phenomenon that is still poorly understood.

Previous studies have identified various factors influencing vaccine uptake in China, such as age, sex, education, self-rated health, perceptions of COVID-19, trust in healthcare workers, and information sources [6, 11, 12]. However, focusing solely on the individual-level determinants may not fully explain the unique vaccination patterns observed among the elderly in China, which likely stem from multifaceted and complicated underpinnings. To comprehend the dynamics of the low elderly COVID-19 vaccination issue in mainland China, it is essential to consider the broader context of politics, policy and culture. Given the inherent connection between COVID-19 vaccinations and political activities, policy changes, and shifts [13], this study applies John Kingdon’s Multiple Streams Framework (MSF), a widely used framework in policy analysis that examines problem, policy, and politics streams throughout the policy process [14]. By employing MSF, this study aims to explore how the macro-level factors shaped the COVID-19 vaccination strategy for the elderly in China during the zero-COVID period. Adopting a retrospective perspective through qualitative content analysis of press conference texts and policy documents, this study seeks to provide a comprehensive explanation for why China adopted a different approach to the prioritization of elderly groups for COVID-19 vaccination compared to many other countries. Furthermore, the study contributes to the understanding of public health policy agenda-setting discussions by examining the specific case of COVID-19 vaccination for the elderly in China.

## Methods

### Data collection

The Chinese government established a Joint Prevention and Control Mechanism (JPCM) of the State Council on January 21, 2020, coordinated by the National Health Commission (NHC). The JPCM involved 32 central government departments, working together to address various aspects of disease prevention and control, medical treatment, scientific research, publicity, and work at the frontline by holding a weekly press conference [15]. The JPCM press conferences served as the authoritative and primary channel for disseminating information about the latest developments, government positions and policy statements regarding COVID-19 to the general public, as well as answering public questions about science and policies. To gain insights into the broader context surrounding COVID-19 vaccination in China, the text of these press conferences, along with relevant policy documents and media coverage, represents a valuable dataset. In this study, we collected 95 press conference transcripts from October 20, 2020 to February 27, 2023, which were available on the official NHC website. Additionally, we gathered COVID-19 vaccination-related policy documents, including the *Protocol for Prevention and Control of COVID-19 (7th-10th ed.)*, *Technical Guideline for the Inoculation of COVID-19 Vaccines*, *Action Plan to Speed Up COVID-19 Immunization of the Elderly, Implementation Plan for Launching Second COVID-19 Booster Immunization* (see details in Appendix 1), which were obtained from official websites of the NHC and local government sources. Media coverage regarding elderly COVID-19 vaccination was also obtained online.

### Data analysis

This study adopts a qualitative approach, employing thematic analysis to analyze press conference transcripts and vaccination policy documents. The analysis was facilitated using NVivo 12. As the data were largely in Chinese, rigorous measures were taken to ensure translation accuracy, including a thorough review of the English translation for any errors or inconsistencies, and consultation with bilingual colleagues to ensure fidelity to the original language. Inductive and deductive approaches were applied to generate themes during the coding process (Table 1). To provide additional context, we annotated each quote with attributes indicating its respective speaker, date, and discourse style. We used a discursive analysis of China’s family planning policy as a reference, which categorized China’s state propaganda into rational (relies on prudent, pragmatic, and calculated arguments to persuade its target audience) and sentimental (deploys affective rhetoric to achieve intuitive, uncalculated, and direct human reactions from the audience that are to the state’s advantage) manners [16]. The study also described the trends in vaccination coverage of people aged 60 years and above based on data obtained from official JPCM press conferences.

**Table 1:** Thematic analysis of the press conference discourse regarding elderly COVID-19 vaccination

Themes	Codes	Descriptions
Vaccination strategy	Vaccination policy	Interpretations and implementations of COVID-19 vaccination policies, such as booster vaccination and sequential vaccination
	Vaccination promotion	Discourse related to vaccination promotion
Vaccination practice	Procedures and notes	Procedures and important notes of vaccination, such as precautions for the elderly, convenience channels for the elderly, and observation time
	Local experiences	Experience sharing of cities/rural areas/nursing homes with good performance
	Problems	Problems occurred through the implementation process of the vaccination program
Vaccine safety	Clinical evidence	Data evidence mentioned to support the general vaccination strategy
	AEFIs and Contraindications	Statements and descriptions of Adverse Event Following Immunization(AEFI) and contraindications

### The theoretical framework

The MSF has been frequently used in the analysis of immunization and healthcare policies [17, 18] and is well-suited for qualitative research. According to Kingdon, the MSF consists of three concurrent but independent streams—problem, policy, and politics—that run through the policy process. The problem stream relates to how and why a condition or an issue is redefined or framed as a problem, the policy stream focuses on the potential solutions to the problem, and the politics stream concerns the broader socio-political context in which policies are influenced and shaped. When the three separate streams converge, a policy window opens, creating an opportunity for significant policy change [14, 19]. Existing studies have successfully used the MSF to analyze the policymaking process within the Chinese context, including epidemic preventions, tobacco control regulations and educational policies, highlighting its applicability in understanding Chinese public policy [20–23]. Thus, we conceptualized the MSF as a theoretical and organizational framework to guide our analysis of the elderly COVID-19 vaccination issue in China. Our focus was on macro-level dimensions of political regimes, ideologies, cultural values, and news media rather than individual-level factors.

### The COVID-19 vaccination context in China

China maintained a strict zero-COVID policy from the onset of the pandemic, enforced by swift lockdowns, mass testing, and travel restrictions to contain outbreaks. In August 2021, the country transitioned to a more flexible “dynamic zero-COVID” strategy to maintain minimal or no indigenous transmission to buy time for vaccination until the population was protected through immunization [24]. On November 11 and December 7, 2022, China announced the “20 Measures” and “New 10 Measures” policies, respectively, which loosened mass testing and quarantine norms and signalling a rapid shift away from the zero-COVID policy [25]. Since then, there has been a nationwide resurgence of infections, which has reignited discussions regarding the necessity and effectiveness of vaccination.

**Fig. 1:**
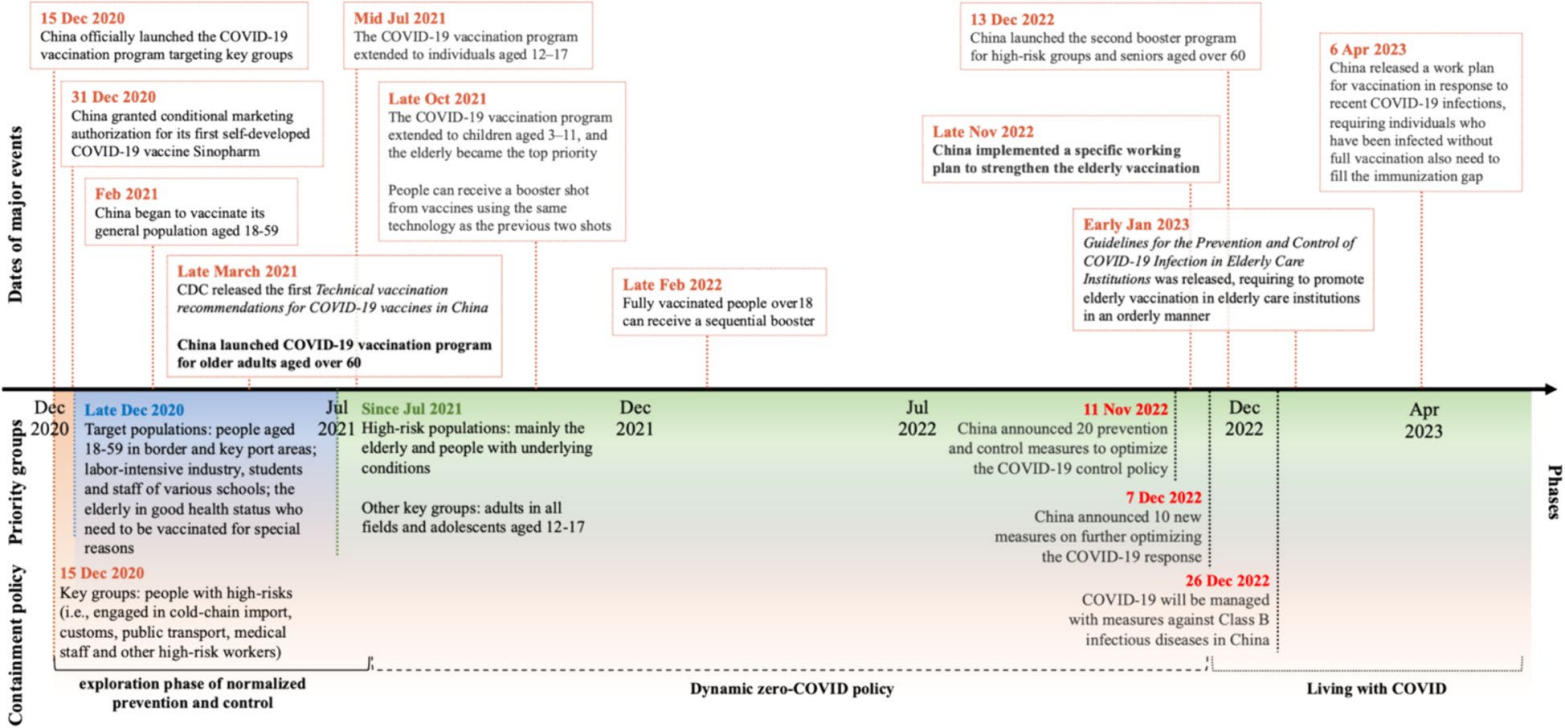
Timeline of the major COVID-19 vaccination policies in China

Looking back over the 3 years pandemic years, China demonstrated its commitment to putting people and their lives first by adhering to the principle of “centered on people.” To accelerate its COVID-19 vaccine research and development efforts, China targeted vaccination to specific populations in a phased and orderly manner in accordance with the “informed, agreed, and voluntary” principle. Based on press conferences and policy documents, we compiled a timeline of major COVID-19 vaccination policies in China (Fig. 1). China initiated the emergency use of COVID-19 vaccines for nearly a million people as early as June 2020, and officially started its mass COVID-19 vaccination program on December 15, 2020. By March 2021, four COVID-19 vaccines for conditional marketing and one for emergency use authorization (with inactivated recombinant protein and adenovirus vector technologies) were approved in mainland China. All COVID-19 vaccines available in China were produced domestically and provided at no cost to Chinese citizens. Seventy percent of the fees for COVID-19 vaccines and vaccination were covered by social health insurance schemes, while the rest was supported by reallocated general tax revenue [26]. As of November 2022, the number of accessible vaccines had increased to seven, and by December 2022, China had 13 COVID-19 vaccines available for a second booster with a mix-and-match approach.

**Fig. 2:**
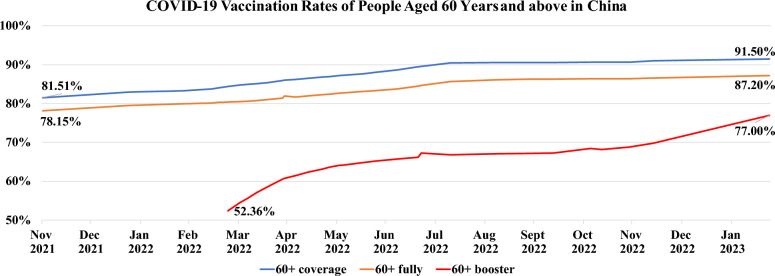
COVID-9 vaccination rates of people aged 60 years and above in China. *Elderly vaccination data were obtained from the official NHC website. To account for variation in reporting, this study used a total elderly population of 264 million (aged 60 years and above) to calculate proportions

China’s top-down, one-party system enabled it to mobilize enormous resources using a “whole-of-society” approach to support COVID-19 vaccination. This approach involved active government engagement in promoting mass vaccination, collaboration among multiple systems and departments from a governance perspective, allocation of sufficient health workers and resources, extensive mobilization and communication for vaccination campaigns, expansion of vaccine financing channels, and implementation of a localized digital information system [26]. Due to these efforts, China reached its first billion dose milestone in June 2021 and had administered over 3.4 billion vaccine doses by July 2021 [27]. Assuming that each person had completed two doses of vaccination, this was sufficient to have vaccinated over 120% of China’s population. Almost all working-age adults had been vaccinated by July 2022. However, despite these achievements, vaccination rates among the elderly sluggishly increased in late July 2022 (Fig. 2).

## Results

### The problem stream

**Fig. 3:**
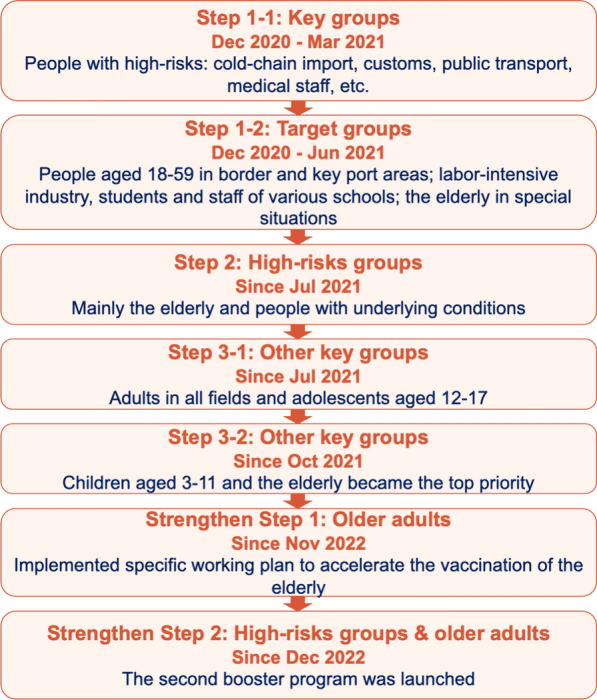
Stages and timeline of mass vaccination rollout

In late 2020, China had good control over the overall COVID-19 situation [28], but continued to face pressure to prevent “external importation and internal rebound.” To address this issue, Deputy Director of the NHC, Zeng Yixin, emphasized that “given the association between recent sporadic outbreaks and imported cold chain logistics, it holds immense importance to prioritize COVID-19 vaccination among key populations, including cold chain logistics personnel and border inspection personnel, who are particularly vulnerable. As the vaccine production and supply assurance capabilities continues to improve, a comprehensive and orderly rollout of vaccination will subsequently extend to other high-risk groups, such as the elderly and individuals with underlying diseases” (JPCM, December 19, 2020). Thus, China first adopted a “step-by-step” national rollout plan for its mass vaccination program, prioritizing several key population groups, including medical staff, customs officers, cold chain workers, and other high-risk workers. Two months later, the program was expanded to include the general adult population aged 18–59 years (see Fig. 3 for details) [29]. Although Beijing and Shanghai piloted vaccinations for some eligible elderly adults and patients with chronic diseases, China did not officially begin vaccinating the elderly aged over 60 years until April 1, 2021, four months after the vaccine rollout had begun. This was in contrast to other countries, which generally placed the elderly as the highest priority for vaccination.

China’s cautious timeline and slow prioritization of COVID-19 vaccination for the elderly may also reflect the lack of safety and efficacy data from clinical trials of vaccines, particularly for the elderly population. The phase III trial of the Sinopharm vaccine had included a relatively small sample of participants aged over 60 years, with only 612 of the 40,411 individuals (1.5%) in this age group [30]. And few older adults in the study had comorbidities. He Qinghua, the first-level inspector of the National Disease Prevention and Control Bureau (NCDC), acknowledged this issue, stating that “large-scale vaccination for the elderly population aged 60 years and above would only be conducted after obtaining sufficient safety and efficacy data in clinical trials” (JPCM, March 21, 2021). Additionally, this cautious approach could also reflect broader Confucian values that prioritize protecting the elderly. Given the low risk of domestic infection under the “Dynamic zero-COVID” strategy, priority was given to key industry personnel and high-risk young people to shield the elderly from contracting the virus. Wu Liangyou, deputy director of the NCDC, explained that “adults over 18 are the main target groups for vaccination, as they are much more mobilized and can establish an effective protective barrier for the elderly and children at home” (JPCM, April 11, 2021). However, this approach, while well intentioned and aligned with deontological values, may have exacerbated the difficulty of encouraging the elderly to vaccinate later on. As people may have adjusted their risk perceptions and vaccination intentions over time, the delay in vaccinating the elderly may have made it more challenging to convince them to get vaccinated [31].

**Fig. 4:**
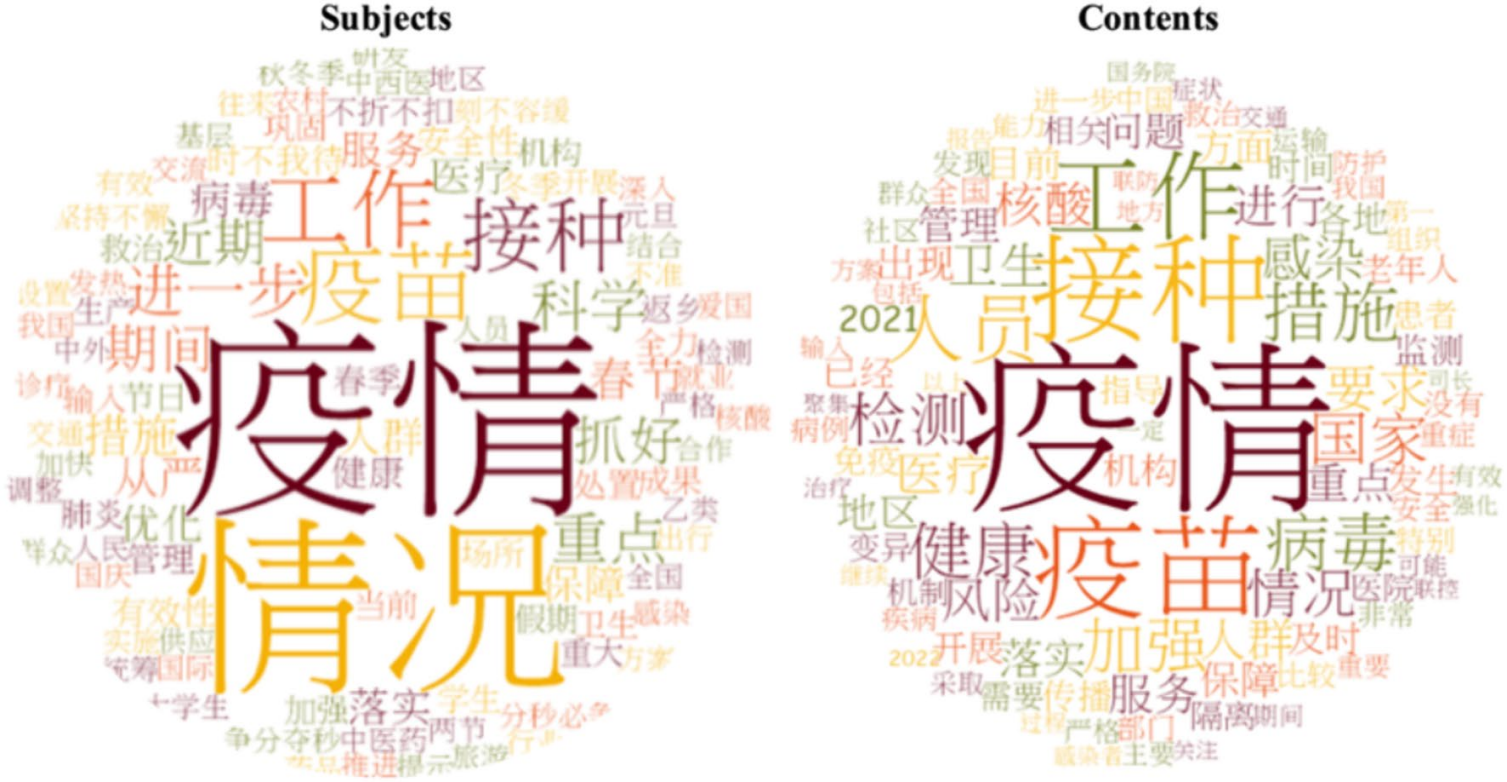
Word frequency of the 95 subjects and content of press conferences

Regarding problem recognition within the problem stream, it is noteworthy that the core of the general dynamic zero-COVID policy was the “rapid elimination of outbreaks when cases are detected,” as emphasized by Liang Wannian, the head of the NHC COVID-19 response expert team, and the key architect of the zero-COVID strategy. While 81 out of 95 press conferences mentioned COVID-19 vaccination-related content (themes listed in Table 1), only 25 of these conferences contained vaccination-related themes in their subjects or were solely centered on COVID-19 vaccination issues. A Word Cloud generated from the subject and content of these conferences also reveals that the frequency of words related to vaccines and vaccinations was less than that of words related to outbreaks and other control measures (Fig. 4). Moreover, as shown in Fig. 1, from late 2021 to mid-2022, despite the inclusion of elderly vaccination status and uptake rates as part of basic reporting and calls to speed up vaccination rates among the elderly at the JPCM press conferences, the issue of vaccinating this population was not a “focusing event” of national policy guidance or implementation.

Despite the issuance of a specific Action Plan to Accelerate COVID-19 Immunization of the Elderly issued on November 29, 2022, the elderly were only prominently mentioned in the “Vaccination” section of the 9th (June 27, 2022) and 10th (January 7, 2023) editions of the Protocol for Prevention and Control of COVID-19, which simply stated, “focus on increasing the full vaccination and booster vaccination rates for those who are at severe high-risk illness, including people aged over 60”. However, these two protocols were primarily focused on infection control, medical treatment, and surveillance. The focus of governance attention on infection control has inadvertently lowered the priority of COVID-19 vaccination, which should be the primary means of prevention and resulted in systematic policy consequences. To address the coming surge of outbreaks resulting from the major policy shift, the “New 10 Measures” policy was issued on December 7, 2022, aimed at optimizing convenient vaccination services, especially for those aged over 80 years. However, promoting vaccination, particularly among the elderly, was challenging at the grassroots level, where high workloads and extended working hours caused fatigue among vaccinators, making them reluctant to serve older adults, especially those with advanced age and underlying diseases [26]. It was difficult to devote significant time and resources to promoting vaccination among the elderly population.

### The politics stream

The Chinese government’s focus on pandemic containment was politically driven, with an emphasis on its superior performance compared to other countries. The success in controlling previous outbreaks fed an ideologically driven complacency toward vaccination, which was an unintended side effect of successful containment, and was observed in other zero-COVID countries [32]. Although China had the state capacity to enforce strict vaccine mandates, as evidenced by the strict Shanghai lockdown, the central government refrained from using coercive political measures to boost vaccine uptake among the elderly. Instead, they manifested the philosophy of “people’s lives first”, which was a distinctive feature of China’s fight against the COVID-19 pandemic. This was mainly due to insufficient clinical data and the complexity of the physical status of the elderly, which made it challenging to determine the most effective mandates for this population. Unlike several Western countries that implemented mandatory vaccination policies with fines for non-compliance, such as Italy (mandates for people over 50) and Greece (fining people aged over 60 who refuse to be vaccinated 100 euros a month) [33, 34], China relied on fewer coercive incentives, such as cash and grocery rewards, as economic nudges to encourage unvaccinated elderly individuals to be vaccinated. Although these soft means broke the international stereotype of China as an authoritarian state, they did not significantly increase vaccine uptake among the elderly during the early rollout period.

During the pandemic, China established an emergency management system with unified leadership, comprehensive coordination, classified management, hierarchical responsibility, and territorial management [26, 35]. From May 2021, COVID-19 vaccination entered the stage of accelerated promotion, with the national teleconference on COVID-19 prevention and control stressing the need “to implement political responsibility and celebrate the centenary of the establishment of the Communist Party of China with extraordinary vaccination rates” (May 5, 2021). However, when accelerating vaccination rates among the elderly, this accountability system proved to be a double-edged sword, making authorities at different levels accountable for political responsibility and the persistent growth of indicators. The discrepancy between central government intentions and local implementation reflected the typical occurrence in China’s top-down political system, where local officials are placed under tremendous pressure to meet policy targets set by the central government.

To encourage elderly vaccination, each region had its own “stick to central” vaccination strategy, although enforcement varied between regions. Simplistic and one-size-fits-all approaches to achieve the “rigid goals” had been reported in many jurisdictions, such as linking vaccination with endowment insurance, medical insurance [36] and restricting access to public venues for unvaccinated people. Although the NHC has repeatedly emphasized that COVID-19 vaccination should follow the “informed, agreed and voluntary” principle and that “simplification or one-size-fits-all approach during the vaccination implementation, which requires all staff to be vaccinated must be rectified”, it has also pinpointed that “individuals who are of the appropriate age and without medical issues should receive COVID-19 vaccination” (JPCM, April 11, 2021). As a result, the elderly fell through the cracks in inconsistent top-down policy implementation.

### The policy stream

Vaccination strategies or guidelines related to vaccine safety, efficacy, and contraindications have evolved with the clinical assessment of risks, vaccination standardization, and severity of local outbreaks. Before March 2021, the JPCM barely mentioned vaccination for the elderly owing to insufficient clinical data. However, in late March 2021, the NHC’s COVID-19 Vaccination Technical Guidelines (1st ed.) listed “chronic diseases under unstable or acute period” as contraindications for vaccination and underlined that “limited phase III clinical trials of the four vaccines with conditional approval for market use” “no data on the protection efficacy of the vaccines” in the vaccination recommendations for people aged 60 and above. Given that seniors are the most vulnerable and most likely to have at least one chronic disease, such as hypertension, diabetes, or even tumors, this broad and ambiguous statement caused concern and confusion about vaccination eligibility among the elderly. This also led to precautious advice from vaccination doctors, which added to the hesitancy of the elderly. Although the guidelines on contraindications and precautions had been updated and made more detailed to avoid ambiguity since April 2021 (see comparisons in Table 2), the initial confusion, variation of information, and overemphasis on risks for those with comorbidities have undermined public opinion and hampered follow-up vaccination campaigns.

**Table 2:** Comparisons of guidelines on contraindications and precautions for COVID-19 vaccination

Guideline version	Eligible conditions (*n*)	Conditions could defer (*n*)	Ineligible conditions (*n*)	Other conditions (*n*)
1st: Apr 2021	29	5	5	14
2nd: May 2021	31	6	6	15
3rd: Sep 2021	33	8	9	15
4th: Jul 2022	33	8	9	14

*Data were obtained from policy documents from the official website of the Heilongjiang Province People’s Government (https://www.hlj.gov.cn)

Since July 2021, China focused its vaccination strategy on “the old and the young.” Due to the highly contagious Delta variant, officials urged eligible people, particularly the elderly, to get vaccinated as soon as possible. The discourse shifted to emphasizing both rational and sentimental encouragement (Table 3). Over time, the style of official discourse had become increasingly sentimental, using the grand concept of “family and country” and repetitive indoctrination instead of rational persuasion. In October 2021, China began delivering a COVID-19 booster vaccination to the public, including people over 60 years of age or with lower immune function. Initially, experts recommended “better use the vaccines produced by the initial vaccine manufacturers or at least choose the vaccines of the same technologies” (JPCM, September 16, 2021). However, four months later, in February 2022, “sequential immunization” was introduced, which encouraged “mixing different technologies of vaccines to boost people’s immune systems and reinforce herd immunity against the disease”. This method gave people a wider choice of boosters. However, although these strategies were all supported by evidence, inconsistent policy content and not yet well-standardized vaccination practices made the elderly hesitant, regardless of the NHC’s call for intensified efforts [37].

**Table 3:** JPCM Discourse of encouraging the elderly to get vaccinated

Time	Speaker	Content	Style of discourse
Aug 27, 2021	Wang Huaqing, Chief Expert of the Immunization Program of NCDC	According to vaccination strategies in other countries and the WHO suggestion, the elderly, especially those with underlying diseases, are one of the top priority groups for vaccination	Rational
Sep 7, 2021	Du Xueping, Director of Beijing Yuetan Community Health Center	The prevalence rate of underlying diseases among the elderly is high, and their own immunity will decrease with age, once infected, they are more likely to develop severe diseases. It is recommended that all healthy, chronically ill but under stable control elderly over 60 should be vaccinated ASAP	Rational
Nov 30, 2021	Zheng Zhongwei, Leader of the Vaccine R&D Task Force of JPCM	Accelerating the promotion of elderly vaccination is crucial for the elderly themselves, their families, and society. Failure to quickly promote it among this 50 million population could lead to a significant number of severe cases and death once control measures are relaxed, thereby severely straining China’s medical resources and causing significant social problems. Only by ensuring a higher uptake of vaccinations among the elderly can China win the initiative and time needed to combat the pandemic	Sentimental
Dec 20, 2021	Wang Huaqing	The COVID-19 vaccine currently used in our country underwent rigorous clinical trials before being approved, including trials for the elderly. The results show that the vaccine is safe and well-tolerated among the elderly	Rational
Jan 22, 2022	He Qinghua, the first-level inspector of the NCDC	We hope that elderly individuals will actively participate in vaccination and fulfill their responsibilities and obligations for pandemic prevention and control, while also ensuring their own health	Sentimental
Feb 26, 2022	Wu Liangyou, deputy director of the NCDC	We urge everyone who is eligible to complete their COVID-19 vaccination and booster vaccination ASAP. Elderly individuals aged over 60, especially those over 80 who have no contraindications, are encouraged to take the initiative to get vaccinated in order to protect their own health and contribute to combating the pandemic	Sentimental
Mar 19, 2022	Zheng Zhongwei	We once again strongly urge the acceleration of elderly vaccinations, especially targeting the older age group, as it is beneficial for individuals, families, society and the country as a whole	Sentimental
May 13, 2022	Wang Huaqing	The incidence of severe illness among the elderly who have received only one dose of an inactivated vaccine and remain unvaccinated is over 20 times higher compared to those who have completed two or three doses of the vaccine, highlighting the importance of full vaccination and enhancing protection against severe illness and death	Rational
Jul 23, 2022	Zheng Zhongwei	Accelerating the vaccination of elderly people is essential to minimize the risk of severe disease and death. It not only benefits the elderly but also takes the initiative in the country’s broader efforts towards epidemic prevention and control	Sentimental
Aug 10, 2022	Wang Huaqing	Numerous studies have shown that the elderly are at a higher risk of contracting COVID-19, leading to severe illness, death, and ICU admission. We recommend that all eligible elderly individuals who have not been vaccinated or have not completed the entire vaccination process, including booster shots, should receive the vaccine as soon as possible. Although vaccination takes some time to provide full protection, it has a significant protective effect and can develop specific immunity among the elderly	Rational
Nov 29, 2022	Xia Gang, Director of the Immunization Department of the NCDC	We aim to provide warm vaccination services to the elderly. We also urge media outlets to play an active role in raising awareness and mobilizing elderly individuals to promptly receive their vaccinations	Sentimental
Dec 13, 2022	Wang Huaqing	Whether living alone or with family, we encourage all elderly individuals to complete the vaccination to obtain better protection and provide peace of mind to their families	Sentimental

In addition to policy content differences and changes in official discourse, social media also shifted its messaging regarding vaccination promotion, especially after witnessing the serious consequences of the fifth wave outbreak in Hong Kong and the outbreak in Shanghai in the first half of 2022. In response to the NHC’s call for intensified efforts, authorities at all levels, health-related institutions, and private media accounts actively encouraged the elderly to be vaccinated. Tendentious words such as “effective,” “expert,” “as soon as possible,” and “safe” appeared more frequently after April 2022 [38]. For example, Shanghai Release, the official account run by the Information Office of Shanghai Municipal People’s Government and the most influential government account in China, published over 60 articles mentioning elderly vaccination between March 25, 2021 (when Shanghai started to vaccinate the elderly) and July 2022. These articles covered topics such as why the elderly should be vaccinated as soon as possible, convenient vaccination services provided (especially for the elderly), risks and benefits, and vaccination rate updates. However, the later rounds of vaccination promotion for the elderly prioritized rushing and achieving infinite growth in vaccine uptake, which had the opposite effect once it reached its extremes. There have been reports of several older adults in Shanxi and Hunan provinces who had already passed away, but their records of COVID-19 vaccination are still updated [39]. This has led to a loss of public faith in local authorities and healthcare facilities.

Furthermore, China leveraged pioneering digital technologies in pandemic containment and management systems, such as health QR codes and vaccine passes [40]. However, older adults often lacked access to or knowledge of modern technology. With low digital literacy, the elderly may not have smartphones and might struggle with electronic vaccination notifications and appointment systems. The high reliance on digital management systems largely overlooked the elderly, forcing them into an even more marginalized and excluded status.

### The policy window

According to the MSF, a policy window is a fleeting opportunity when the convergence of the problem, politics, and policy streams occurs; a problem is recognized, political impetus arises, and the policy solution is appropriately developed and implemented [14]. However, during the zero-COVID period in China, the three streams did not converge, and China did not seize the opportunity window to accelerate vaccination when limited outbreaks and cases occurred. Initially, China did not prioritize the elderly due to its focus on infection control rather than prevention, insufficient clinical evidence, and broader Confucian values that younger generations should shoulder the responsibility of protecting the elderly. Low and late attention has delayed the promotion of vaccination among the elderly (problem stream). Meanwhile, the whole society was ideologically complacent, adopting soft enforcement measures to encourage the elderly to be vaccinated (politics stream). Additionally, ambiguous statements of contraindications and precautions, inconsistent policy content, radical message-shifting on social media, and a less age-friendly digital management system influenced the intentions of the elderly for vaccination (policy streams). Consequently, the vaccination process for the elderly was undermined, compelling the most vulnerable to be the Achilles’ heel of China’s COVID-19 prevention and control strategy.

In December 2022, China relaxed its COVID-19 containment measures. However, despite the high vaccination rates among the general population, a surge in cases could not be prevented. Once again, China focused on extinguishing the pandemic and accelerating medical treatment, resulting in a lower priority for promoting vaccination, particularly among the elderly. Wang Huaqing also emphasized that “individuals who test positive for COVID-19 should not receive COVID-19 vaccination in the near future” (JPCM, January 14, 2023). Later, in 2023, as the haze of the pandemic gradually faded and life returned to normal, China issued a Work Plan for Vaccination against Recent COVID-19 Infection on April 6, which sought a sustainable exit from the pandemic. However, the previous strong public opposition caused the central government to lack the will and proper measures to implement a new vaccine pass. Due to repeatedly missing the policy window, the opportunity to compensate for the lost time had passed, and elderly vaccination rates in China have not yet reached satisfactory levels.

## Discussion

Building on the MSF, this study analyzes the underpinnings of the sluggish vaccination rates of the Chinese elderly by examining the three streams within the framework. This study demonstrates that China missed the optimal policy window to promote elderly vaccination due to the poor convergence of the three streams.

### Reflections on the MSF application in China

Most existing public health literature uses the MSF to analyze why a particular health issue has successfully garnered policy attention and featured on the public health agenda [17–19]. However, there is relatively less research that examines why certain issues fail to feature prominently on the agenda [22, 41]. Nonetheless, both types of research seek to understand the dynamics of issue framing and policy agenda-setting and how they can be effectively addressed and adopted. Our study contributes empirical insights to the latter and adds to the MSF literature given the unique demographic and political context of China during the zero-COVID period, which is often considered non-democratic and authoritarian.

Unlike previous analyses on HPV vaccination issues in the US and Canada, where policy entrepreneurs and interest groups play critical roles in shaping policy outcomes [18, 42], the influence of policy entrepreneurship in the third sector is comparatively limited in the policymaking process within the Chinese context. Instead, the primary policy entrepreneurs in China tend to be experts with close connections to the government or those directly involved in government and affiliated think tanks. The Chinese government mobilizes resources such as COVID-19 vaccine manufacturers and non-government organizations (NGOs) to drive policy changes in a top-down approach. This observation aligns with research by Van den Dool and Schlaufer on policy process theories in autocratic systems, which argues that participation in policymaking is restricted in authoritarian regimes. Due to institutional constraints, NGOs and the general public play only a minor role in policy processes [23]. Additionally, while existing studies often conceptualize each stream as independent and separate from the others, our study found that in China, the three streams, especially the policy and politics streams, are unavoidably interconnected and influenced each other (e.g., vaccine pass, health codes, etc.), with some policy responses serving certain political goals (such as decentralized tasks of vaccination rates). This interplay among the streams is likely to emerge in a broader socio-political environment where the fundamental zero-COVID policy is highly political, further highlighting the significant role of politics in shaping public health policies in China.

### Learning from the elderly COVID-19 vaccination in China

Strategies for COVID-19 containment have developed differently across countries and areas owing to different COVID-19 situations, political regimes, cultural values, levels of sociodemographic development, and governance capacity. COVID-19 vaccination policies and priority populations have also been developed over time. In terms of the problem stream, the issue of elderly vaccination in China did not receive sufficient and foresighted attention initially, as the priority was drastic containment measures and low mortality rates, with timely suppression of the outbreak rather than COVID-19 vaccination. A previous study also concluded that China’s current emergency response policy for public health emergencies pays more attention to the prevention and control of public emergencies, and less attention to prevention measures [43].

In national vaccination programs, the typical approach is to prioritize the maintenance of essential functions of society and the protection of high-risk populations, such as individuals with underlying conditions and older adults aged 60 years and above [44, 45]. However, in the case of China, instead of prioritizing the elderly, the younger generation was given precedence in receiving vaccinations, with the intention of building up an immune barrier to protect the older population. This decision was influenced by the deep-rooted deference given to the elderly in Chinese culture, as well as the uncertainties surrounding clinical evidence at the time. This initial precautious approach towards prioritization hampered the uptake of COVID-19 vaccines among the Chinese elderly population. In contrast, Japan, which also values Confucian principles with the world’s super-aged population, prioritized vaccination for its elderly and achieved successful vaccination growth in the short term, leading to a decline in infections among the elderly during outbreaks [46]. These divergent policy decisions underscore the inherent challenges in setting priorities for target groups within vaccination programs and the need to optimize resources to ensure vaccine coverage based on ethical, scientific, and practical considerations when determining vaccine prioritization [47, 48].

Zhou stressed the critical role of political processes, organizational structure, attention allocation, and decision timing in shaping policy outcomes when analyzing the multiple decision-making processes in the early stages of the pandemic in the US [49]. Similarly, our study on the low vaccination rate among the elderly in China shows the significant impact of attention and timing on the policymaking process. China’s empirical experience indicates that problem recognition and priority assessment should allow flexible adjustment according to changes in the actual situation, especially during a pandemic with huge environmental uncertainty.

In the politics stream, social scientists suggested that governments need to address people’s concerns and barriers to vaccination when complacency is a factor in driving vaccine demand [32]. However, in China, the government has not responded effectively to people’s concerns or hesitancy. Additionally, while the Chinese government aimed to break the stereotype of authoritarian governance at the central level by using the general principle of voluntary vaccination and soft enforcement measures, local governments had to adopt simplistic and one-size-fits-all enforcement measures to pursue the “Chinese speed” of vaccination under political pressure. This has created a constant dilemma for vaccination strategies such as a swinging pendulum.

In the policy stream, previous studies have shown that appropriate and unprecedented policies can be enacted despite changing evidence or evidentiary uncertainty, particularly when addressing upstream factors that influence health [50]. A study in Singapore found that the uncertainty brought about by the pandemic resulted in inconsistencies in the government’s public health messaging, which has influenced the public’s trust in the government [51]. Likewise, our study indicates that policy information should be clearly disseminated to the public through consistent messaging to avoid distrust of specific policies. Moreover, although the elderly are given full respect and afforded the autonomy to make decisions about their own health choices in accordance with the general voluntary principle [52], implicit ageism in the implementation of vaccination policies should not be ignored. In the current context where governments emphasize the importance of digital management to contain the pandemic, such “average” benefits of digital technologies and systems for a population can impose individual inconveniences on the vulnerable population [53]. Attention should also be paid to this ageism-like issue when implementing policies, and technology should be developed and deployed in a way that is accessible and beneficial to all members of society, particularly older adults.

Although authoritarian governments are more likely to impose strict censorship on major news media to control the flow of news and information to the public [35], our study aligns with a previous study on China’s policymaking of tobacco control, which reveals that even in a controlled environment, the power of mainstream media through the policymaking process still fits the general roles proposed by the international community, serving as information disseminators, social mobilizers, and problem warners [20]. Regarding vaccination information, the media should function as a better disseminator, focusing more on changing content and providing consistent and scientific explanations instead of solely simplistic promotion. The public needs time to digest the policies to act in response, and message framing and shifting should be gentle and gradual rather than adopting an aggressive manner. This will help build trust in the government and encourage compliance with critical policies aimed at addressing the COVID-19 pandemic.

### Limitations

Vaccination is always a trade-off between the risks and benefits. While individuals make decisions based on scientific evidence and rational considerations, this article explores how macro-environmental factors, such as politics and policies, influenced elderly vaccination in China during the pandemic. Since the study is descriptive in its analysis of qualitative materials, we acknowledge several limitations. First, due to certain constraints, the researchers were unable to conduct interviews with policymakers and high-level key informants regarding elderly COVID-19 vaccination policies in China. Thus, the analytical perspective of the study relies solely on official and public sources. The absence of insights from insiders may limit the depth of understanding regarding the decision-making processes and considerations involved. Second, the analysis mainly drew on JPCM press conferences and policy documents from December 2020 to February 2023, while the COVID-19 vaccination strategy is expected to evolve continuously against new variants in the future. Therefore, the current analysis and findings are limited to this particular timeframe. Further research is required to explore the long-term effects and implications of vaccination policies on the elderly population in China and other countries.

## Conclusion

This study explored the underpinnings of the sluggish vaccination rates among the elderly in China through the lens of the MSF. The analysis implies that China missed the optimal policy window to encourage vaccination among the elderly due to the poor convergence of the problem, policy, and political streams. This study emphasizes the need for future research to explore priority identification; enforcement measure adoption; and timely, appropriate, and effective policy dissemination. The empirical lessons from the COVID-19 vaccination of the elderly in China could inform the crafting and facilitation of future vaccination policies and have broader implications for rethinking policy responses in preparation for the next major public health crisis.

## Data Availability

The data supporting the findings of this study are publicly available from press conferences, government documents, media platforms, etc.

## Appendix 1

**Table:**

No.	Date	Document name	Publisher
1	Apr 6, 2020	Technical Guidelines for the Prevention and Control of COVID-19 Infections in Medical Institutions (Edition 2)	NHC
2	Sep 11, 2020	Protocol for Prevention and Control of COVID-19 (Edition 7)	JPCM of the State Council
3	Mar 29, 2021	Technical Guideline for the Inoculation of COVID-19 Vaccines (Edition 1)	NCDC
4	Apr 1, 2021	COVID-19 Vaccination Q&A (March 31, 2021 Update)	NHC
5	Aug 1, 2021	Guidelines for COVID-19 Control and Protection in Key Places, Units, and Populations under Normal Conditions (August 2021 Update)	JPCM of the State Council
6	May 11, 2022	Protocol for Prevention and Control of COVID-19 (Edition 8)	JPCM of the State Council
7	Jun 27, 2022	Protocol for Prevention and Control of COVID-19 (Edition 9)	JPCM of the State Council
8	Nov 11, 2022	Notice on Further Optimizing COVID-19 Prevention and Control Measures to Implement Scientific and Precise Prevention and Control Work	JPCM of the State Council
9	Nov 29, 2022	Action Plan to Speed up COVID-19 Immunization of the Elderly	JPCM of the State Council
10	Dec 7, 2022	Notice on Further Optimizing the Implementation of COVID-19 Prevention and Control Measures	JPCM of the State Council
11	Dec 9, 2022	Action Plan for Health Services for Key Populations in COVID-19 Prevention and Control	JPCM of the State Council
12	Dec 13, 2022	Action Plan for Implementing Booster Doses of COVID-19 Vaccines for Strengthening Immunity	JPCM of the State Council
13	Dec 15, 2022	Action Plan for Strengthening COVID-19 Prevention and Control and Health Services in Rural Areas	JPCM of the State Council
14	Dec 24, 2022	Notice on Adjusting the Target Population for Vaccination with Zhifei Longcom Recombinant COVID-19 Vaccine (CHO Cells) and Implementing Booster Doses	JPCM of the State Council
15	Dec 26, 2022	Overall Plan for Implementing Class B Infectious Diseases Management of COVID-19 Infections	JPCM of the State Council
16	Jan 7, 2023	Protocol for Prevention and Control of COVID-19 Infection (Edition 10)	JPCM of the State Council
17	Jan 7, 2023	Guidelines for COVID-19 Infection Prevention and Control	JPCM of the State Council
18	Apr 6, 2023	Action Plan for COVID-19 Vaccination in Response to Recent Infections	JPCM of the State Council
